# Supplementary material for: Molecular Cloning and Functional Analysis of 1-Deoxy-D-Xylulose 5-Phosphate Reductoisomerase from Santalum album
Source: Genes (Basel). 2021 Apr 22;12(5):626. doi: 10.3390/genes12050626 (PMC8143465; doi:10.3390/genes12050626)
Supplement: Supplementary file 1 [file genes-12-00626-s001.zip › genes-1164086-supplementary.pdf]

Supplementary Table 1. Primers used in this paper

Primer name	Primer Sequences 5' → 3'	Usage
GSPF1	TTGTGATTCATCCGCAGTC	3' RACE
GSPF2	ATCTTGCCTATGCTGCTGG	3' RACE
GSPR1	CACCTGGTCAGCAAGTAGAGTCACATTA	5' RACE
GSPR2	GCAACTATGTCCAATGTCTGAGTTCCAA	5' RACE
GSPF3	TAGGGCAAGCAGTGGTATCAACGC	Full-length
GSPR3	CTACCACTCATACGGGAACAGGGCT	Full-length
gDNAF	CTCTGAGCTTATCGAGATTTCTCTAGGC	gDNA
gDNAR	ACTCATACGGGAACAGGGCTCAAT	gDNA
FP1	GTAATACGACTCACTATAGGGCACGCGTGGT NTCGASTWTSGWGTT	1st PCR primer for FPNI-PCR
FP2	GTAATACGACTCACTATAGGGCACGCGTGGT NGTCGASWGANAWGAA	1st PCR primer for FPNI-PCR
FP3	GTAATACGACTCACTATAGGGCACGCGTGGT WGTGNAGWANCANAGA	1st PCR primer for FPNI-PCR
FP4	GTAATACGACTCACTATAGGGCACGCGTGGT AGWGNAGWANCAWAGG	1st PCR primer for FPNI-PCR
FP5	GTAATACGACTCACTATAGGGCACGCGTGGT NGTAWAASGTNTSCAA	1st PCR primer for FPNI-PCR
FP6	GTAATACGACTCACTATAGGGCACGCGTGGT NGACGASWGANAWGAC	1st PCR primer for FPNI-PCR
FP7	GTAATACGACTCACTATAGGGCACGCGTGGT NGACGASWGANAWGAA	1st PCR primer for FPNI-PCR
FP8	GTAATACGACTCACTATAGGGCACGCGTGGT GTNCGASWCANAWGTT	1st PCR primer for FPNI-PCR
FP9	GTAATACGACTCACTATAGGGCACGCGTGGT NCAGCTWSCTNTSCTT	1st PCR primer for FPNI-PCR
FSP1	GTAATACGACTCACTATAGGGC	2nd PCR primer for FPNI-PCR
FSP2	ACTATAGGGCACGCGTGGT	3rd PCR primer for FPNI-PCR
FPNIR	GGTTGTGCTGAACAACACAGTATT	1st PCR primer for FPNI-PCR
FPNIR1	TTGGACTCTGGGAAGGTGGATG	2nd PCR primer for FPNI-PCR
FPNIR2	GCCTAGAGAAATCTCGATAAGCTCAG	3rd PCR primer for FPNI-PCR
YFP-Bgl II-F	GGAAGATCTTAATGGCTCTGAATTCGCTCTCT	YFP vector construction
YFP-Kpn I-R	GGGTACCGTACGGGAACAGGGCTCAAT	YFP vector construction
qRTSaActin1F	CAGCCATACAGTGCCCATATAC	qRT-PCR
qRTSaActin1R	CCTTCATGTCCCTCACGATTT	qRT-PCR
qRTAtActin2F	GGAATCCACGAGACAACCTATAAC	qRT-PCR
qRTAtActin2R	TGGACCTGCCTCATCATACT	qRT-PCR
qRTSaSSyF	GCTTGTGGATGTGGTTCAAAG	qRT-PCR
qRTSaSSyR	CACTGTCGTAGCAGCCTAAA	qRT-PCR
qRTSaDXRF	ACTCCACCAAGCCCATCCACCT	qRT-PCR
qRTSaDXRR	GCTGAACAACACAGTATTCTCC	qRT-PCR
qRTAtDXRF	GATACCTCCAGGTTCAATCCAATC	qRT-PCR

Supplementary Table 1 Continued		
qRTAtDXRR	GAGGAGGTTGTTGTTGCTGCTG	qRT-PCR
SemiRTSaDXRF	ACTCCACCAAGCCCATCCACCT	Semi-quantitative RT-PCR
SemiRTSaDXRR	CTGATTTCTAACGGCCACTAGC	Semi-quantitative RT-PCR
SemiRTAtActin2F	GGTAACATTGTGCTCAGTGGTGG	Semi-quantitative RT-PCR
SemiRTAtActin2R	TGGACCTGCCTCATCATACT	Semi-quantitative RT-PCR
1302-Nco I-F	GGACTCTTGACCATGGTAATGGCTCTGAATTC GCTCTCT	Overexpression vector construction
1303-Nco I-R	GTCAGATCTACCATGGTTACGGGAACAGGGC TCAAT	Overexpression vector construction

Supplementary Table 2. The exons and introns in *SaDXR*, *AtDXR*, *PtDXR* and *VvDXR*

	Length in nucleotides			
	SaDXR	*AtDXR*	*PtDXR*	*VvDXR*
Exon 1	88	91	88	88
Intron 1	1227	196	317	1545
Exon 2	176	194	179	179
Intron 2	290	154	461	363
Exon 3	87	87	87	87
Intron 3	241	235	173	397
Exon 4	126	126	126	126
Intron 4	86	65	84	85
Exon 5	63	63	63	63
Intron 5	940	95	317	990
Exon 6	150	150	150	150
Intron 6	134	117	199	223
Exon 7	67	68	68	68
Intron 7	518	93	381	476
Exon 8	113	112	112	112
Intron 8	104	86	113	110
Exon 9	105	105	105	105
Intron 9	165	86	167	122
Exon 10	122	122	122	122
Intron 10	771	288	317	580
Exon 11	147	147	147	147
Intron 11	334	85	293	342
Exon 12	172	169	169	169
